# Metals and metal mixtures associated with the odds of uterine fibroid prevalence among African Caribbean women in the Tobago Health Study

**DOI:** 10.3389/frph.2026.1917571

**Published:** 2026-08-17

**Authors:** Alexis Kiyanda, Hoimonty Mazumder, Iva Miljkovic, Ryan Cvejkus, Janet Catov, Victor Wheeler, Patrick J. Parsons, Elizabeth J. Mullin, Austin A. Roberts, Aaron Barchowsky, Natalie Price, Alison P. Sanders

**Affiliations:** 1 Department of Environmental and Occupational Health, University of Pittsburgh School of Public Health, Pittsburgh, PA, United States; 2 Department of Epidemiology, University of Pittsburgh School of Public Health, Pittsburgh, PA, United States; 3 Tobago Health Studies Office, Scarborough, Trinidad and Tobago; 4 Division of Environmental Health Sciences, Wadsworth Center, New York State Department of Health, Albany, NY, United States; 5 Department of Environmental Health Sciences, College of Integrated Health Sciences, University at Albany, Albany, NY, United States

**Keywords:** arsenic, Caribbean, environmental health, metal exposure, reproductive health, Tobago, uterine fibroid

## Abstract

Uterine fibroids are the most common benign smooth muscle neoplasm of the uterine wall and are a major indication for hysterectomy. Although common risk factors include African Ancestry, family history, obesity, and early menarche, exposure to non-essential metals is an emerging risk factor that has not been previously examined in Caribbean populations. In this cross-sectional study, a sample of the prospective Tobago Health Study, we assessed associations between individual urinary concentrations of metals and metal mixtures with self-reported uterine fibroid prevalence among 441 women (40–87 years old). We used logistic regression to estimate associations between concentrations of six metals (non-essential metals—arsenic, cadmium, and lead; essential metals—copper, manganese, and zinc) individually and as a mixture with the odds of uterine fibroids. Models were adjusted for urinary creatinine, age, body mass index, education, and number of live childbirths. We assessed the effects of metal mixtures using weighted quantile sum regression. The rate of prevalence of uterine fibroids was 48.3%. In adjusted models, each 10-fold increase in µg/L urinary concentrations of total arsenic, dimethylarsinic acid, and arsenobetaine was associated with 61%, 84%, and 38% higher odds of uterine fibroids, respectively (*β*: 1.61; 95% CI: 1.04, 2.53); (*β*: 1.84; 95% CI: 1.11, 3.09); and (*β*: 1.38; 95% CI: 1.05, 1.82). Other metals were not associated with the odds of uterine fibroids. We observed no significant associations between metal mixtures and the odds of uterine fibroids. Urinary arsenic and arsenical metabolites were associated with higher odds of uterine fibroids in a cohort of African Caribbean women. Raising awareness about arsenic and metal exposure is prudent, and further research in longitudinal studies is needed to replicate the findings of this study and inform policies for public health protection of women's health.

## 1 Introduction

Uterine leiomyoma, or fibroids, are benign smooth muscle neoplasms of the uterine wall that can develop in more than 70% of women of reproductive age (1, 2). Symptomatic fibroids can result in lower quality of life and are the leading cause for hysterectomy (2, 3). Although the causes of uterine fibroids are still unknown, common risk factors include family history, obesity, poor diet, and early menarche (1, 2). In addition, people of African descent (as observed primarily through research in the United States) are disproportionately affected by uterine fibroids with a 2–3 times higher risk compared with those who are white, including fibroids that are larger in size and quantity, and these occur at an early age (4–7). The 2023 Global Burden of Disease (GBD) review examined the global scope of uterine fibroids and found that Caribbean and Sub-Saharan African regions with lower reported fibroid prevalence and incidence rates were likely underestimated because of discrepancies among uterine fibroid diagnoses (8–11).

Exposure to non-essential metals is associated with detrimental reproductive conditions in individuals with a uterus, including irregular menstruation, birthing complications, and reproductive cancers (12–15), while the links between essential metals and reproductive health are less clear. Low-level concentrations of non-essential and essential metals may be ingested through drinking water and certain diets (e.g., a high seafood intake may have higher exposure to arsenic, cadmium, or lead), inhaled as metal-containing dusts or particulate matter, or absorbed dermally via personal care products such as makeup (12–14). Exposures to essential metals such as copper, manganese, and zinc also occur through ingestion from dietary and drinking water sources as well as inhalation of airborne particulates (12–15). Exposure to some elements may occur through household objects (e.g., lead in paint and toys and arsenic in pesticides), jewelry (e.g., copper and arsenic), or personal care products (e.g., arsenic, cadmium, and lead in makeup) (12, 16). Six prior epidemiologic studies explored whether low-level exposures to arsenic, cadmium, or lead were associated with an increased risk of uterine fibroids, with several suggesting links to uterine fibroid prevalence (17–22). Two of these studies also reported inconclusive but potential associations with essential metals copper, manganese, and zinc (20, 21). Metal-dysregulated pathways may play a role in the pathobiology of uterine fibroids, given that estrogen is necessary for the development of fibroid growth, and a complex milieu of hormonal, chemical, genetic, and epigenetic factors that dysregulate estrogen receptor functioning is well-supported in the literature (23–30).

Both essential and non-essential metal exposures are especially relevant to populations residing in the Caribbean region, where lifestyle factors and industrialization from mining, waste disposal sites, and petrochemical plants may reflect unique exposure profiles (31–36). Our previous work on the Tobago Health Study (THS) showed that women's urinary cadmium and zinc levels were higher than the levels observed in similar global population–based studies (37). Only a few prior studies have examined the links between metal exposure and uterine fibroids in African Caribbean women despite a disproportionately higher risk of incidence and the size of uterine fibroids (5, 6). Therefore, in this study, we assessed the cross-sectional associations between urinary concentrations of individual metals (including total arsenic and speciated arsenic) and metal mixtures with uterine fibroid prevalence in the THS. We prioritized toxic metals as well as essential metals with suggested evidence of epidemiologic relationships (17–22, 31). We hypothesized that non-essential metalloid/metals such as arsenic (and arsenical metabolites), cadmium, and lead would be associated with higher odds of uterine fibroids, while essential metals such as copper, manganese, and zinc would be associated with lower odds.

## 2 Methods

### 2.1 Study population

The Tobago Women's Health Study, part of the Tobago Health Study, is a population-based study of the role of body composition and related risk factors in driving cardiometabolic diseases among women aged 40 years and older (38). The study collected health history and spot urine samples between 2019 and 2020 through visits at the Tobago Health Office in Scarborough. A total of 1180 women participants were recruited by word of mouth within the healthcare setting using protocols described in previous studies (38, 39). To be eligible, women had to be non-institutionalized, ambulatory, and not terminally ill. Urinary metal concentrations were measured in spot urine samples for a random subsample of 466 women. Analyses excluded participants without data on self-reported uterine fibroid prevalence and number of live childbirths (*n* = 25). Thus, a subcohort of 441 participants with complete information on the history of uterine fibroid(s) and urinary metal measurements was included in the analyses (Supplementary Figure S1). Written informed consent at enrollment was obtained from all participants. The study was approved by the University of Pittsburgh and the Tobago Division of Health, Wellness and Social Protection Institutional Review Boards.

### 2.2 Assessment of urinary non-essential and essential metals

Urine samples were analyzed for a panel of 18 trace elements, including arsenic—a metalloid, but hereafter, they are collectively referred to as “metals.” We selected six metals *a priori* for our analyses from 13 of the metals with sufficient detection [>60% of samples above the limit of detection (LOD)] (37). Selection of the six metals was based on suggested evidence from epidemiological or toxicological literature for their putative relationships to fibroid development or prevalence (17–22, 26–28, 31, 40). Spot urine samples were obtained in the morning, frozen at −80°C, and batch-shipped to the University of Pittsburgh for storage. Urinary concentrations of arsenic, cadmium, lead, copper, manganese, and zinc were quantified in participant urine samples (2-mL aliquots) at the Wadsworth Center's Human Health and Exposure Analysis Resource (HHEAR) at the New York State Department of Health. The Wadsworth Center is a certified laboratory improvement amendments (CLIA)-certified laboratory and NIH-designated HHEAR lab hub with quality assurance protocols for quantifying trace elements in human biospecimens (41).

Trace elements, including total urinary arsenic, were measured in urine samples using inductively coupled plasma tandem mass spectrometry (Agilent Model 8900 ICP-MS/MS) equipped with an Octopole Reaction System and axial acceleration technology. The instrument was configured with an SPS 4 autosampler (Agilent Technologies, Santa Clara, CA, USA) with an ultralow particulate arrester air filter installed (Elemental Scientific, Omaha, NE, USA) to minimize airborne contamination during analysis. Non-creatinine-adjusted urinary metal concentrations were reported as µg/L. For analytes where metal concentrations were below the LOD, an imputed value of LOD/sqrt(2) replaced the instrument reported values (42). Urinary creatinine (mg/dL) was also measured primarily as an indicator of hydration status and included as a covariate in the main models; the results using creatinine-standardized metal concentrations are reported in Supplementary Materials.

Arsenic speciation was performed using high-performance liquid chromatography (HPLC) coupled to ICP-MS/MS to separate five arsenic species [inorganic arsenic (iAs), i.e., ∑As^3+^ and As^5+^, dimethylarsinic acid (DMA), monomethylarsonic acid (MMA), arsenocholine, and arsenobetaine]. Urinary arsenicals and metabolites that represent exposure to inorganic arsenic sources (∑iAsM) were calculated as the sum of iAs, DMA, and MMA concentrations (iAs + DMA + MMA). A complete description of the arsenic speciation method used in the Tobago Health Study was provided by Jahan et al. (43) As part of a sensitivity analysis, we examined arsenobetaine, DMA, iAs, MMA, and ∑iAsM in a subset of 440, 439, and 352 participants with arsenic speciation measurements. Arsenocholine was detected in fewer than 42% of samples and not included in statistical analyses.

### 2.3 Assessment of uterine fibroid prevalence and covariate selection

Standardized interviewer-administered questionnaires were used to obtain information on ethnicity, education, current smoking and alcohol intake, and personal and family health history. History of uterine fibroid(s) was self-reported by participants as the binary variable of (Y/N). Participant information regarding age of menarche, ever/never pregnant, and number of live childbirths was also self-reported. Body mass index (BMI) in kg/m^2^ was calculated from measured body weight and standing height (kg/m^2^) at the time of the study visit. Self-reported highest education was categorized into nine levels that ranged from primary school to receiving a doctoral degree using the questionnaire information on “what is the highest grade or level of education that you completed?” Our analysis classified participants into three educational groups: (1) No formal education or primary school; (2) Secondary school O and A, and vocational school; and (3) At least some college to having a doctoral degree. Information about smoking and alcohol use was measured but not included in analyses because of low prevalence within the study population.

### 2.4 Statistical analyses—associations between metals and fibroids

We used logistic regression to estimate the associations between log_10_-transformed urinary metal concentrations and the odds of uterine fibroids. Models were adjusted for covariates such as urinary creatinine, age, BMI, highest education attained (three groups), and number of live childbirths. Furthermore, age of menarche and ever/never pregnant were not included in our final model because of multicollinearity with the number of live childbirths. In exploratory subanalyses, we also performed adjusted logistic regression of individual arsenic species such as arsenobetaine and DMA (*n* = 440), iAs (*n* = 439), and MMA and ∑iAsM (*n* = 352). The resultant odds ratio effect estimates (ORs) were converted to a percentage increase in odds using [(OR-1) x 100%] and interpreted as a reduction in odds for ORs < 1.0 or increase in odds for ORs > 1.

We used weighted quantile sum (WQS) regression to evaluate the association between metal mixtures and the odds of uterine fibroids (44). WQS indices were simultaneously estimated for both positive and negative directions, allowing for identification of mixture components associated with increased or decreased odds of uterine fibroids. Each metal was transformed into quartiles, and the dataset was randomly split into training (40%) and validation (60%) sets, where mixture component weights are estimated and WQS indices are estimated, respectively. This 40%/60% split is consistent with WQS convention allocating the larger split to the validation set to increase power for the hypothesis-testing step (45). Component weights were estimated via 100 bootstrapped samples in the training set, while the positive and negative WQS indices were calculated in 100 repeated holdout iterations in the validation set adjusted for covariates (random seed=123). Final estimates reflect the average WQS regression coefficients and exposure weights across all repetitions, with uncertainty summarized using confidence intervals. This bidirectional extension of WQS relaxes the same-direction assumption of the original WQS framework by estimating two separate sets of component weights, one for the positive index and one for the negative index simultaneously, rather than constraining all components to contribute to the mixture effect in a single direction (46).

As a secondary analysis, we used Bayesian Kernel Machine Regression (BKMR) to evaluate potential joint, non-linear effects of the metal mixture with the odds of uterine fibroids. BKMR was implemented using a Gaussian Kernel and the model was run for 50,000 Markov Chain Monte Carlo (MCMC) iterations with the first 10,000 discarded as burn-in. Non-linear relationships and interactions between mixture components were assessed visually using univariate and bivariate exposure-response plots from the BKMR package (47).

We also conducted supplemental analyses using urine creatinine-standardized metal concentrations in μg/g, calculated by dividing metal concentration in μg/L by creatinine (mg/dL) and multiplying by a conversion factor of 100 mg/g. In supplemental creatinine-standardized models, we adjusted for identical covariates as above, with the exception that urinary creatinine was not included. In addition, three arsenic methylation efficiency indices reported as ratios (e.g., MMA/iAs; DMA/MMA; and MMA/DMA) were calculated to reflect individual detoxification capabilities.

## 3 Results

### 3.1 Participant demographics and descriptive statistics of metals

In this random subcohort of 441 African Caribbean women participating in the Tobago Women's Health Study, the mean age was 55.6 ± 8.6 years (range: 40–87 years old) and the mean BMI was 31.0 ± 5.7 kg/m^2^. The prevalence rate of uterine fibroids was 48.3% (Table 1). The mean age of menarche was 12.8 ± 1.8 years, 391 participants (92.5%) had at least one live birth, and the mean number of live childbirths was 2.6 children per participant. With regard to highest education attained, 129 participants (29.2%) completed primary school; 223 participants (50.6%) completed secondary school O or A, or vocational school; and 89 participants (20.2%) obtained at least some college or higher. Participant characteristics are reported in Table 1.

**Table 1:** Characteristics of Tobago Women's Health Study participants with information on uterine fibroid history (*n* = 441).

Characteristic		All participants (*n* = 441)	Participants with uterine fibroids (*n* = 213)	Participants without uterine fibroids (*n* = 228)
Age (years)		55.6 ± 8.6	55.8 ± 7.7	55.4 ± 9.4
BMI (kg/m^2^)		31.01 ± 5.67	31.12 ± 5.65	30.91 ± 5.70
Age of menarche (years)		12.8 ± 1.8	12.8 ± 1.8	12.9 ± 1.9
Pregnancies, *n* (%)				
Never pregnant		33 (7.5)	24 (11.3)	9 (3.9)
Ever pregnant		408 (92.5)	189 (88.7)	219 (96.1)
Number of live births, categorical, *n* (%)				
Never delivered an infant		50 (11.3)	33 (15.5)	17 (7.5)
Ever delivered an infant		391 (88.7)	180 (84.4)	211 (92.5)
Parity per individual		2.6 ± 1.8	2.3 ± 1.7	2.9 ± 1.9
Nulliparous, *n* (%)		50 (11.3)	33 (15.5)	17 (7.5)
Multiparous, *n* (%)		391 (88.7)	180 (84.5)	211 (92.5)
Highest education attained (3 analysis categories), *n* (%)				
No formal education, primary school		129 (29.2)	54 (25.4)	75 (32.9)
Secondary school O, secondary school A, and vocational Training		223 (50.6)	111 (52.1)	112 (49.1)
At least some university, university graduate, master's degree, and doctoral degree		89 (20.2)	48 (22.5)	41 (18.0)
Metal concentrations in urine (µg/L)				
Non-essential metals	Arsenic (total)^a^	34.7 ± 2.98	38.5 ± 2.87	31.5 ± 3.06
	Arsenobetaine (*n* = 440)^b^	13.0 ± 5.60	15.9 ± 4.99	10.8 ± 6.11
	DMA (*n* = 440)^b^	8.67 ± 2.82	9.50 ± 2.76	7.95 ± 2.87
	MMA (*n* = 352)^c^	1.19 ± 2.42	1.27 ± 2.27	1.12 ± 2.54
	iAs (*n* = 439)^d^	0.625 ± 1.96	0.624 ± 1.91	0.626 ± 2.02
	∑iAsM (*n* = 352)^c^	9.02 ± 2.39	9.56 ± 2.28	8.56 ± 2.49
	Cadmium	0.734 ± 2.11	0.762 ± 2.01	0.708 ± 2.21
	Lead	0.857 ± 1.74	0.884 ± 1.73	0.833 ± 1.75
Essential metals	Copper	13.8 ± 1.80	13.3 ± 1.77	14.2 ± 1.82
	Manganese	0.268 ± 2.05	0.271 ± 1.97	0.266 ± 2.12
	Zinc	627 ± 2.27	596 ± 2.22	658 ± 2.31
Creatinine concentration in urine (mg/dL)^e^		149 ± 1.75	147 ± 1.73	152 ± 1.76
Alcohol intake, *n* (%)				
0 drinks/week in past 12 months		378 (85.7)	184 (86.4)	194 (85.1)
[1,4) drinks/week in past 12 months		55 (12.5)	28 (13.1)	27 (11.8)
[4,7) drinks/week in past 12 months		4 (0.9)	1 (0.5)	3 (1.3)
7 or more drinks/week in past 12 months		4 (0.9)	0 (0.0)	4 (1.8)
Smoking status, *n* (%)				
Never		428 (97.0)	209 (98.1)	222 (97.4)
Past		6 (1.4)	2 (0.9)	4 (1.8)
Current		7 (1.6)	2 (0.9)	4 (1.8)

Data are presented as frequency (%) or mean ± standard deviation, except for metals, which are presented as geometric mean ± geometric standard deviation. BMI, body mass index.

a Total arsenic was quantified by ICP-MS and is not expected to equal the arithmetic sum of individually speciated arsenic compounds, as the two measurements were obtained by independent analytical methods with differing scopes and detection capabilities.

b Arsenobetaine and DMA were analyzed in a subset of 440 participants with available data.

c Summed arsenicals and arsenic metabolites were analyzed in a subset of 352 participants with available data.

d Inorganic arsenic was measured in urine from a subset of 439 participants with available data.

e Urine creatinine was used to calculate standardized metal concentrations as shown in Supplementary Table S2.

Geometric mean concentrations of the selected metals are presented in Table 1, and complete descriptive statistics are presented in Supplementary Tables S1, S2 (for creatinine-standardized metals). The geometric mean concentration of the non-essential metals, cadmium and lead, was 0.734 ± 2.11 and 0.857 ± 1.74 µg/L. The geometric mean concentrations of the total arsenic, ∑iAsM, arsenobetaine, DMA, MMA, and iAs were 9.02 ± 2.39, 13.0 ± 5.60, 8.67 ± 2.82, 1.19 ± 2.42, and 0.625 ± 1.96 µg/L. The geometric mean concentrations of the essential metals of copper, manganese, and zinc were 13.8 ± 1.80, 0.268 ± 2.05, and 627 ± 2.27 µg/L. A Wilcoxon rank-sum test was used to compare metal urinary concentrations of participants with and without fibroids; total arsenic and arsenobetaine were the sole elements where concentrations differed between participants with fibroids and participants without fibroids (*W* = 21,529, *p* = 0.040); (*W* = 21,178, *p* = 0.025). Distributions of the non-essential and essential metals are shown in Supplementary Figure S2. Three arsenic methylation efficiency indices were higher among participants with fibroids than those without fibroids, but only the primary arsenic methylation efficiency index of MMA/iAs was significantly higher comparing participants with fibroids with those without (ratio: 2.10 ± 1.71; ratio: 1.79 ± 1.78, *p* = 0.014) (Supplementary Tables S1, S2).

### 3.2 Individual non-essential and essential metals and the odds of fibroids

**Figure 1:**
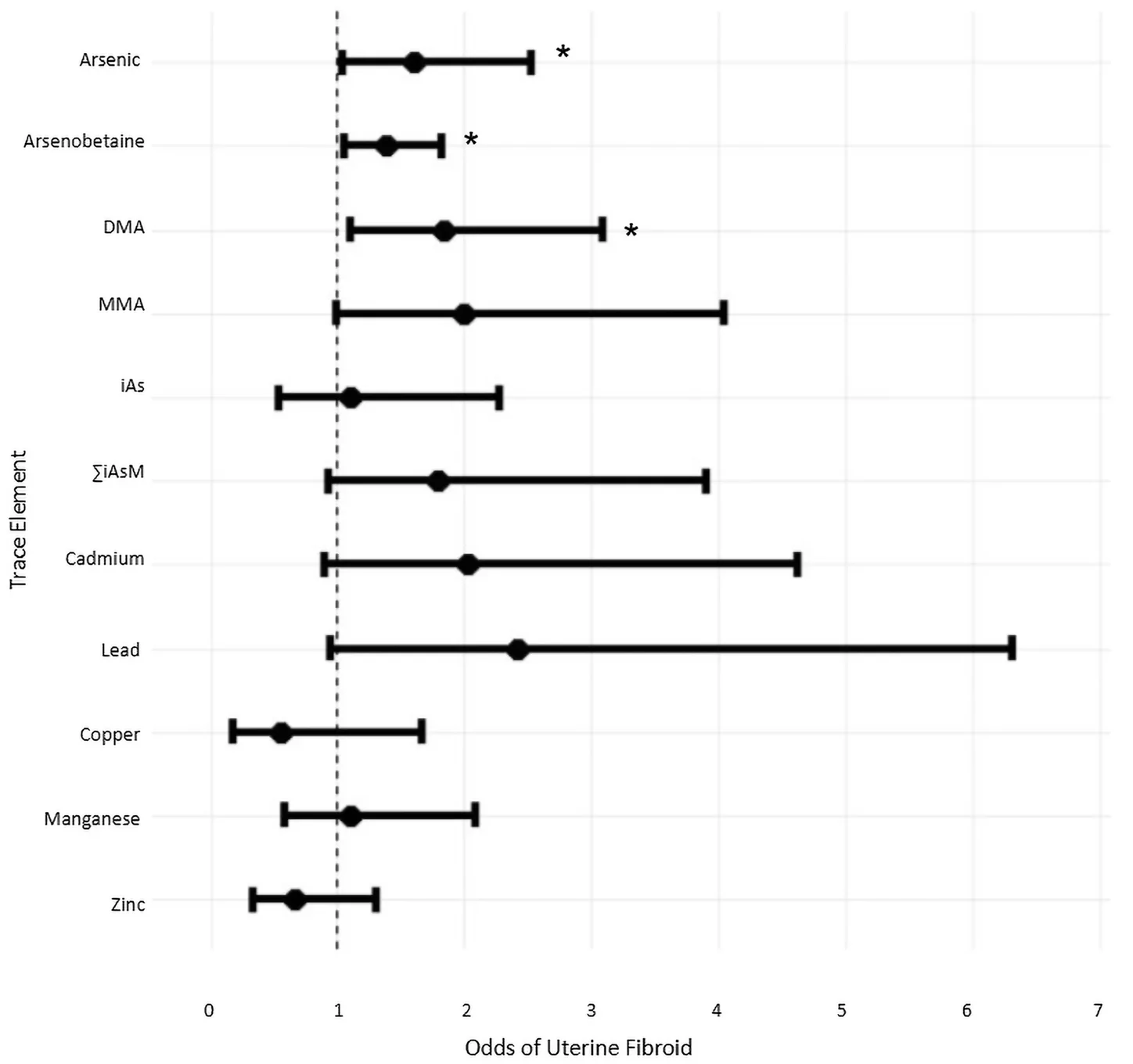
Odds ratios (ORs) of uterine fibroids by metal in adjusted models. Each 10-fold increase in µg/L urinary concentration of arsenic, DMA, and arsenobetaine corresponded to a 1.61 (95% CI: 1.04, 2.53), 1.84 (95% CI: 1.11, 3.09), and 1.38 (95% CI: 1.05, 1.82) higher odds of uterine fibroids. *p < 0.05.

In adjusted models, each 10-fold increase in the urinary concentration (µg/L) of total arsenic corresponded to a 1.61 (95% CI: 1.04, 2.53) higher odds of uterine fibroids (Table 2, Figure 1, Supplementary Table S3). Cadmium and lead were not significantly associated with the odds of uterine fibroids; the ORs for cadmium (OR: 2.03; 95% CI: 0.90, 4.63) and lead (OR: 2.42; 95% CI: 0.95, 6.31) were suggestive of a possible positive association. The essential metals copper, manganese, and zinc were not significantly associated with uterine fibroids, but the direction of effect for copper and zinc was consistent with our hypothesized protective effect. In further analyses of arsenicals, DMA and arsenobetaine were associated with 1.84 (95% CI: 1.11, 3.09) and 1.38 (95% CI: 1.05, 1.82) higher odds of uterine fibroids, respectively. Summed urinary arsenicals (∑iAsM), iAs, and MMA were not associated with the odds of uterine fibroids. We compared our findings with those of prior studies including study design (17–22), population characteristics, metal concentrations, and effect estimates (Table 3).

**Table 2:** Adjusted odds ratios of uterine fibroid prevalence by individual metal and metabolites.

Metal		Adjusted model OR (95% CI), *n* = 441
Non-essential metals	Arsenic (total)	1.61 (1.04, 2.53)^d^
	Arsenobetaine^a^	1.38 (1.05, 1.82)^d^
	DMA^a^	1.84 (1.11, 3.09)^d^
	MMA^b^	1.99 (0.99, 4.02)
	iAs^c^	1.11 (0.54, 2.28)
	∑iAsM^b^	1.89 (0.93, 3.90)
	Cadmium	2.03 (0.90, 4.63)
	Lead	2.42 (0.95, 6.31)
Essential metals	Copper	0.55 (0.18, 1.67)
	Manganese	1.11 (0.59, 2.09)
	Zinc	0.67 (0.34, 1.31)

a Arsenobetaine and DMA were analyzed in a subset of 440 participants with available data.

b Summed arsenicals and arsenic metabolites of MMA were analyzed in a subset of 352 participants with available data.

c Inorganic arsenic was analyzed in a subset of 439 participants with available data.

d Metals and metabolites associated with uterine fibroids with *p* < 0.05.

**Table 3:** Comparison of THS findings with prior epidemiological studies that examined the association between metal concentrations and uterine fibroids.

Study design and location	Population and size	Age range in years	Geometric mean metal concentrations and biological matrix among participants with fibroids	Geometric mean metal concentrations and biological matrix among participants without fibroids	Finding
Cross-sectional Seoul, Korea (18)	288 women (46 with fibroids, 242 without)	30–49	Cadmium_blood_: 1.07 ± 1.523 µg/L Lead_blood_: 1.43 ± 1.43 µg/dL, or 14.3 ± 14.3 µg/L	Cadmium_blood_: 0.97 ± 1.43 µg/L Lead_blood_: 1.35 ± 1.41 µg/dL, or 13.5 ± 14.1 µg/L	Blood concentrations in µg/L of cadmium and µg/dL of lead were 104% and 29% higher in women with uterine fibroids compared with controls
Case–control Owo, Nigeria (22)	100 women (50 with uterine fibroids and 50 controls)	20–50	Cadmium_blood_^a^: 0.028 ± 0.02 mg/L, or 28 ± 20 μg/L Arsenic_blood_: 0.040 ± 0.02 mg/L, or 40 ± 20 μg/L Lead_blood_: 0.089 ± 0.04 mg/L, or 89 ± 40 μg/L	Cadmium_blood_: 0.005 ± 0.00 mg/L, or 5 ± 0 μg/L Arsenic_blood_: 0.006 ± 0.00 mg/L, or 6 ± 0 μg/L Lead_blood_: 0.014 ± 0.01 mg/L, or 14 ± 10 μg/L	Each standard-deviation increase in the µg/L blood concentrations of cadmium, arsenic, and lead was associated with 162%, 2%, and 42% higher odds of uterine fibroids
Cross-sectional 14 US sites (17)	473 women (99 with uterine fibroids)	Mean age of 32	Cadmium_whole blood_: 0.37 μg/L Lead_whole blood_: 0.83 μg/dL, or 8.3 μg/L	Cadmium_whole blood_: 0.30 μg/L Lead_whole blood_: 0.61 μg/dL, or 6.1 μg/L	Each standard deviation increase in µg/L or μg/dL concentration of whole blood cadmium and lead was associated with 44% and 31% higher odds of uterine fibroids, respectively. Urinary metals were not associated with the odds of uterine fibroids
Case–control China (21)	30 women (15 with hysteromyoma or “barrenness”	Not reported*			Serum concentrations of copper were significantly higher in individuals with uterine fibroids than in controls, while lower zinc levels were observed in individuals with uterine fibroids
Cross-sectional US NHANES (19)	4,502 women (542 with uterine fibroids, 3,960 without)	Mean age of 34	Lead_whole blood_^b^: 0.053 (0.041,0.082) µmol/L, or 1.10 µg/dL Cadmium_whole blood_: 3.560 (2.580,5.870) nmol/L, or 0.40 µg/L	Lead_whole blood_^b^: 0.048 (0.034,0.071) µmol/L, or 1.10 µg/dL Cadmium_whole blood_: 3.560 (1.780,5.340) nmol/L, or 0.40 µg/L	In whole blood, each standard deviation increase in the quartile log-transformed nmol/L cadmium concentration and µmol/L lead concentration was correlated to uterine fibroid prevalence
Prospective cohort Detroit, Michigan through SELF study (20)	1,132 women without uterine leiomyomata (UL) at baseline, 117 developed UL after a 20-month follow-up	23–35	Cadmium_blood_: 0.23 ± 2.39 μg/L Arsenic_blood_: 0.33 ± 2.56 μg/L Lead_blood_: 0.50 ± 1.63 μg/L Copper_blood_: 901 ± 1.23 μg/L Manganese_blood_: 6.71 ± 1.40 μg/L Zinc_blood_: 4,924 ± 1.21 μg/L		Increasing whole blood µg/L concentrations of cadmium from the 25th to 75th percentile was positively associated with uterine leiomyomata
Cross-sectional Scarborough, Tobago through THS Study	441 women (213 with uterine fibroids, 228 without)	40–87	Arsenic_urine_: 38.5 ± 2.87 µg/L Arsenobetaine_urine_: 15.9 ± 4.99 µg/L DMA_urine_: 9.50 ± 2.76 µg/L Cadmium_urine_: 0.762 ± 2.01 µg/L Lead_urine_: 0.884 ± 1.73 µg/L	Arsenic_urine_: 31.5 ± 3.06 µg/L Arsenobetaine_urine_: 10.8 ± 6.11 µg/L DMA_urine_: 7.95 ± 2.87 µg/L Cadmium_urine_: 0.708 ± 2.21 µg/L Lead_urine_: 0.833 ± 1.75 µg/L	Each 10-fold increase in urinary total arsenic, DMA, and arsenobetaine concentration (µg/L) was associated with 61%, 84%, and 38% higher odds of uterine fibroids

a Study reported median ± standard deviation.

b Study reported median and IQR.

### 3.3 WQS and BKMR analyses

We did not observe significant associations between metal mixtures and the odds of uterine fibroids in either positive or negative direction (Supplementary Tables S4, S5). For the WQS index constrained to be positive, every quartile increase in the pWQS index was associated with 33% higher odds of uterine fibroids; the relationship was not significant but suggestive of a possible positive association (*β*: 1.33; 95% CI: 0.92, 1.93). Similarly, for the WQS index constrained to be negative, every quartile increase in the nWQS index was associated with 5% lower odds of uterine fibroids; however, the relationship was also not significant (*β*: 0.95; 95% CI: 0.62, 1.44). Although neither the positive nor the negative WQS indices were significant, the estimated component weights for each metal mostly aligned with the hypothesized direction of effect (Supplementary Figure S3).

In BKMR analyses, we did not observe any evidence of non-linear relationships of metals with the odds of uterine fibroids nor the interaction effects of metals (Supplementary Figures S5, S6). Although the univariate exposure-response function for some metals appeared visually non-linear, the 95% credible intervals did not exclude a linear fit across the range of exposure.

## 4 Discussion

### 4.1 Summary of key findings

We found that higher urinary concentrations of total arsenic and some arsenic species were associated with higher odds of uterine fibroids in the THS cohort. A 10-fold increase in the concentration of total arsenic, DMA, and arsenobetaine were associated with 61%, 84%, and 38%, respectively, higher odds of uterine fibroids. We cautiously interpret the associations with urinary arsenobetaine because it is primarily considered a non-toxic indicator of fish consumption (48). Yet, the observed findings for both arsenobetaine and the toxic metabolite DMA contributing the greatest magnitude of effect of total arsenic exposure are deserving of further study. Copper, manganese, zinc, cadmium, and lead were not associated with the odds of uterine fibroids, and we observed no significant associations between metal mixtures and uterine fibroids. However, we note that associations with higher urinary concentrations of cadmium, lead, manganese, and MMA, although not statistically significant, were potentially suggestive of higher odds of uterine fibroid prevalence. Similarly, we observed that higher urinary concentrations of copper and zinc were suggestive of lower odds of uterine fibroids; although not statistically significant, these estimates were consistent with the expected direction of effect. The null WQS findings in both the positive and negative directions may suggest independent effects of individual metals or may reflect limited statistical power, given the smaller number of mixture components and/or sample size, rather than a true absence of joint effects.

### 4.2 Comparison with prior epidemiological studies

The associations of arsenic exposure with higher odds of uterine fibroids reported herein add to six prior population-based epidemiological (cross-sectional, case–control, and prospective cohort) studies of metal exposure and fibroids, as shown in Table 3. In a case–control study of 100 reproductive-aged women (50 with uterine fibroids and 50 age-matched controls) in Nigeria, each standard-deviation increase in the µg/L blood concentrations of cadmium, arsenic, and lead was associated with 162%, 2%, and 42% higher odds of uterine fibroids (ORs: 2.62, 1.02, and 1.42, respectively) (22). In a case–control study of 473 reproductive-aged Black women (99 with uterine fibroids) across 14 US sites, each standard deviation increase in the µg/L concentration of whole blood cadmium and µg/dL concentration of blood lead was associated with 44% and 31% higher odds of uterine fibroids (17). In a cross-sectional study of 288 premenopausal women (46 with fibroids and 242 without) in Seoul, South Korea, Ye et al. reported that increases of blood cadmium and lead concentrations were associated with a 104% and 29% higher odds of uterine fibroids in unadjusted models; however, the relationship was no longer significant when adjusted for covariates (18). Two studies, a cross-sectional study of US women in NHANES and SELF, a prospective cohort study of Black women in Detroit, MI, also reported similar magnitude of associations between increasing levels of blood cadmium and lead with higher odds of uterine fibroids (19, 20). Our findings that total arsenic was associated with 61% higher odds of uterine fibroids is a similar magnitude effect size observed in prior studies; moreover, our study observed associations with speciated arsenic including DMA and an 84% higher odds of uterine fibroids, which have not been previously reported. Although, to our knowledge, no epidemiological studies have investigated arsenic metabolite DMA in relation to uterine fibroids, an NHANES study identified associations with higher urinary concentrations of MMA and endometriosis, potentially suggesting that arsenic metabolites may be associated with reproductive tissues more broadly (49). Arsenobetaine and DMA may not be independent and the presence of both in populations with high seafood consumption often complicate the evaluation of the health effects of inorganic arsenic and its main metabolites, MMA and DMA (50). A recent systematic review showed no risk from arsenobetaine on a range of health effects (51). However, arsenobetaine was recently shown in Kagawa et al. to be the main arsenic metabolite associated with hypertension in a Japanese population with high fish meat intake (52).

Our results also suggest a putative relationship between individual cadmium and lead concentrations with higher odds of fibroids, as observed in prior studies. When compared with similar global population–based studies, THS women had a similar geometric mean of urinary arsenic, lead, and copper, while cadmium and zinc levels were among some of the highest reported (37). However, there are no global studies that examine urine metals and fibroids across several populations.

There have been a few epidemiological studies that examined associations between essential metals (e.g., copper, manganese, or zinc) with uterine fibroids. In a small case–control study of 30 Chinese women, He et al. reported that higher serum concentrations of copper were significantly higher in individuals with uterine fibroids in comparison with controls, while lower serum zinc levels were found in the group with uterine fibroids (21). In contrast, the SELF cohort study of Black women in Detroit, MI did not observe associations with blood measures of copper and manganese nor zinc and uterine fibroids (20). However, our study investigated urinary measures of essential metals, given their putative antioxidant and/or apoptosis-mediating contributions to fibroid reduction or prevention.

Our findings are difficult to compare directly with prior studies that assessed metals in blood rather than urine. We note that urine lead and cadmium measures likely indicate cumulative metal exposure burden, whereas urine arsenic measures reflect recent exposures, which may explain the differences reported in our study compared with prior studies that nearly used all blood samples as the biological matrix for metal assessment. Other factors that could contribute to differences in the effect sizes and OR estimates could be differences in age and menopause status between cohorts. Age is associated independently with metal concentration and uterine fibroid status (1, 2). The average age of our cohort was 55 years old, and the average age of menopause in individuals of African Descent is 48 years (53, 54). Cohorts of participants with different age-associated hormonal milieu including menopause status (i.e., pre-, peri-, and postmenopause) may have varied metal toxicokinetics, which could contribute to heterogeneity in observed associations across studies (55–57).

Geographic and dietary variation in metal exposure not only contributes to heterogeneity across studies, but also highlights the need for population-specific biomonitoring data. For example, prior studies in Tobago reported higher concentrations of inorganic arsenic in imported and domestic chicken meat and seafood, which are widely consumed in Tobago (35, 36). A recent review article by Boda et al. reported that several Latin American and Caribbean (LAC) countries were missing adequate biomonitoring of toxic metals such as lead (58). LAC countries that have implemented monitoring policies report that even relatively low blood lead levels are associated with adverse developmental and reproductive health issues. Thus, our work addresses a key gap in reproductive health and environmental exposure biomonitoring in LAC countries.

### 4.3 Pathophysiological relevance and hypothesized mechanisms of toxicity

Briefly, prior *in vivo* and *in vitro* studies have suggested potential pathophysiological mechanisms by which exposure to non-essential metals such as cadmium and arsenic may contribute to uterine fibroid growth. *In vitro* uterine leiomyoma cell models have explored signaling pathways of uterine fibroid growth considered “non-canonical,” or indirectly related to estrogen-receptor dysregulation (26, 27). For example, an *in vitro* study of cadmium-exposed human uterine leiomyoma cells found that altered epidermal growth factor receptor (EGFR) signaling may promote uterine fibroid formation, while another study found that EGFR signaling was induced by the transactivation of the G protein-coupled estrogen receptor following cadmium exposure in human uterine leiomyoma cells (26, 27). Other studies have explored arsenic in the context of *in vivo* mouse models and *in vitro* mouse muscle progenitor cells. A study employing an *in vivo* mouse model examined increased epithelial proliferation in mice uteri after 28-day arsenic exposure periods (28). A study using an *in vitro* model of mice muscle progenitor cells determined that arsenic exposure activated EGFR signaling to dysfunctional tissue differentiation and proliferation (29). An *in vivo* rat study indicated that arsenic exposure decreased downstream uterine estrogen receptor signaling pathways and potentially induced oxidative stress by generating reactive oxygen species (40). Thus, these studies indicate that arsenic and cadmium exposure may plausibly contribute to dysregulation of pathways associated with fibroid growth. However, the relevance of estrogen-mediated metal toxicity is uncertain, given the probable postmenopause profile of our study population. We cannot determine whether study participants’ metal concentrations, measured after fibroid diagnosis, were representative of long-term exposure burden during the period of fibroid development, nor whether an estrogen-related mechanism was involved at that time.

Recent studies suggest that arsenobetaine, considered a marker of dietary arsenic intake from seafood, may be partially converted into more toxic metabolites including DMA; however, a potential mechanism is unknown. A study by Kagawa et al. demonstrated increased hypertension in mice that were fed a human equivalent amount of arsenobetaine in their diet, suggesting that arsenobetaine may have unrecognized health effects in mammals (52). Arsenobetaine has biological effects in marine species (52, 59), suggesting a need for epidemiological and mechanistic studies to fully investigate arsenobetaine effects on proliferative tissue disorders such as uterine fibroids. Thus, careful consideration of dietary seafood is needed to assess these putative pathways. Our study was unable to evaluate associations with potential dietary sources of arsenicals.

In an *in vivo* teleost fish model, lead exposure was implicated in dysregulated estrogen receptor signaling through the measurement of decreased plasma estrogen concentrations (60). It is possible that lead exposure may contribute to downstream dysregulated estrogen pathways implicated in fibroid growth. Yet, to our knowledge, no prior mechanistic studies of fibroids examined metal mixture effects. In contrast to non-essential metals, the mechanistic evidence linking essential metals to uterine fibroid pathobiology is more limited, although there are some plausibly related pathways such as decreased manganese concentrations, decreased antioxidant capacity, and increased oxidative stress (67–70). To our knowledge, there have been no prior toxicologic studies that examined zinc or copper exposures with *in vivo* or *in vitro* models of uterine fibroids or uterine cells. However, these mechanisms are speculative due to our inability to differentiate endogenous and exogenous essential metal concentrations within our study.

More research is needed to untangle how metal-dysregulated pathways may play a role in the pathobiology of uterine fibroids, particularly studies of uterine epithelial, myometrial, or stromal cells.

### 4.4 Strengths and limitations

A major strength of this study is that it is the first to study metals, speciated arsenicals, and metal mixture exposures and uterine fibroid prevalence in an African Caribbean cohort. Furthermore, the THS is a well-characterized cohort of African Caribbean women, an understudied population in not only fibroid research but also environmental and biomedical research. The relatively large and random sample size of 441 African Caribbean women was sufficient to detect significant differences in statistical analyses. Also, the urinary metal analysis followed stringent HHEAR protocols for QA/QC using validated methods, ensuring high accuracy in urinary metal measurements to inform our results (41).

Although there are several strengths in this study, there are a few limitations of note. A temporal limitation is that the diagnosis of uterine fibroids was done before the exposure measurement and metal concentrations at the time of fibroid development and diagnosis were unknown. We were unable to assess causality because a retrospective assessment of uterine fibroid history and urinary metal measurements was carried out after diagnosis. Within the study, uterine fibroid history was self-reported retrospectively and was not evaluated or validated using ultrasound or histological assessment, leading to potential outcome misclassification. Because a large proportion of people affected by fibroids may be asymptomatic (2–4, 7), it is necessary to have validated measures of ultrasounds to accurately determine fibroid status. However, bias associated with self-reporting would likely result in non-differential outcome misclassification and attenuation bias of results toward the null. The likelihood of fibroid detection is associated with access to healthcare, health-seeking behaviors, and socioeconomic status, which may also influence exposure to metals and metalloids (5, 9, 61). This may lead to differential detection biases among participants with access to proper diagnostic tools and resources. In addition, age at fibroid development was unknown, and we were unable to distinguish between pre- and postmenopause fibroid development, which may be important in this aging population. Although the average age of participants was 55 years among participants with and without uterine fibroids, we could only assume that many THS participants were postmenopause because menopause status was not confirmed. The absence of menopause status and information on age at diagnosis meant that there could be menopause-status-related differences in metal toxicokinetics that we were unable to assess (56). Furthermore, urine as a matrix for metal exposure is best suited for measuring the long-term burden of cadmium and lead exposures, while the urinary measurements of arsenic reflect more recent exposures. We emphasize the temporal limitations of this analysis, including that urinary arsenic, indicative of recent exposure, was not assessed during the etiologically relevant window of fibroid initiation. We acknowledge the limitation that urinary creatinine reflects muscle mass, renal function, and nutritional status, and that its role as a covariate may not be purely a dilution correction (71). Our study was unable to determine which participants had stable arsenic exposures from more consistent dietary intake such as seafood, further leading to attenuation bias where recent arsenic exposure could over-represent true, lifetime arsenic exposure. The presence of the essential metals in urine could reflect dysregulated homeostasis and may not adequately reflect dietary intake (67, 68). Furthermore, residual confounding in our statistical analyses may exist because of unmeasured covariates such as vitamin D concentrations, social determinants of health, diet, and number of fibroids (4, 56, 62–64).

### 4.5 Recommendations for future studies

Future epidemiologic studies should include speciated arsenic assessment, detailed ascertainment of fibroid history, collection of date of fibroid diagnosis, measured vitamin D concentration, and validated measures of fibroid diagnosis, such as fibroid severity indicative of grade, size, and number (55, 56, 61–63). Longitudinal study designs are necessary to evaluate temporal associations and make causal inferences about the relationship between metal exposure and uterine fibroids, which we were unable to do in this cross-sectional study. In future work, exposure assessment should employ appropriate biomarkers, matrices, and appropriately timed collections (e.g., speciated arsenic in urine to assess exposure) to contribute to causal inference. Additional mechanistic research is needed to assess individual metals, arsenicals (e.g., DMA, MMA, and arsenobetaine), and mixture effects on uterine epithelial smooth muscle cell and fibroblast responses, and how metal exposures affect cell proliferation, cancerous behaviors, or growth relating to uterine fibroids. Further assessment of speciated arsenicals for associations with uterine fibroids must be prioritized, given that there are sex differences in arsenic metabolism, particularly for DMA, wherein women may be at risk of greater reproductive health implications (65, 66). Studies that examine how uterine endothelial, myometrial, and epithelial cells are exposed to varied hormones, with or without the addition of a metal (or metals), will also be key to elucidating pathophysiological mechanisms that are relevant to different critical stages of normal hormone fluctuations across a lifetime (e.g., reproductive years vs. pregnancy vs. postmenopause). Future studies will better identify the temporality of these relationships and inform potential interventions or policies to protect women from non-essential metal exposure associated with uterine fibroid prevalence.

### 4.6 Conclusions and public health implications

Our study builds upon and supports the limited prior epidemiological findings that arsenic, particularly DMA, and possibly cadmium and lead exposure may contribute to uterine fibroid development. Our cautiously interpreted findings support suggestive associations between urinary total arsenic, DMA, and arsenobetaine concentrations indicative of recent exposures with higher odds of uterine fibroids among Tobagonian women. Similar to other studies, we also observed suggestive trends of long-term exposure burden for cadmium and lead with higher odds of uterine fibroids and zinc and copper with lower odds. Raising awareness of non-essential metal exposure is prudent and can inform guidance and policies to protect women's health. This study contributes to enhancing the research representation of Tobagonian and other African Caribbean populations, who are typically underrepresented, and supports the body of literature addressing the effects of non-essential metals in fibroid etiology.

## Language use and person-centered terminology

Our study refers to study participants as “women” to remain consistent with the wording used in the Tobago Health Study clinical research forms, given that questions regarding gender identity were not asked at the time of data collection. This decision reflects efforts to adhere to local norms meaningful to our participants, as inquiries of gender identity could be perceived as culturally insensitive and cause participant discomfort. However, we use more inclusive language such as “individuals with a uterus” when referring to larger, more broad global communities.

## Positionality statement

Author AK identifies as a Black cisgender woman of Caribbean descent and a public health researcher; her personal and academic identities are deeply intertwined in this work. AK approaches this research not only as a scientist, but as someone whose community is disproportionately affected by uterine fibroids.​ AK's interest in the associations between environmental exposures and reproductive health outcomes in African Caribbean women is rooted in both a cultural commitment to community wellness and a critical understanding of the systemic inequities that shape health risks. AK recognizes that her perspective is shaped by lived experience, and she carries both the privilege and responsibility of bringing these narratives into academic spaces.

## Data Availability

The datasets presented in this study can be found in online repositories. The names of the repository/repositories and accession number(s) can be found below: Metal concentration data for this analysis are hosted and available through the Human Health Exposure Resource (HHEAR) Data Repository (https://hheardatacenter.mssm.edu), which has been approved under the Icahn School of Medicine at Mount Sinai IRB Protocol #16-00947. Access to other study population data is available upon request, pending submission of an analysis plan and approval by the Tobago Health Study.

## Glossary

- Uterine fibroids are benign smooth muscle neoplasms of the uterus.
- Inductively coupled plasma tandem mass spectrometry (ICP-MS/MS) measures the concentration of metals and other analytes.
- Weighted quantile sum (WQS) analyses assess the weights of individual metals in metal mixture models after several iterations of training and validation, bootstrapping, and model fitting.
- Primary school refers to levels 1st through 5, typically for children ages 5–11 years old.
- Secondary school O refers to high school, formal grades 1st–5th. Typically, this is for children aged 11–16 years old.
- Secondary school A refers to high school, formal grade 6th. Typically, this is for children aged 16–18 years old.
