# Supplementary material for: Metals and metal mixtures associated with the odds of uterine fibroid prevalence among African Caribbean women in the Tobago Health Study
Source: Front Reprod Health. 2026 Aug 17;8:1917571. doi: 10.3389/frph.2026.1917571 (PMC13526914; doi:10.3389/frph.2026.1917571)
Supplement: Supplementary file 1 [file Datasheet1.pdf]

Metals and metal mixtures associated with odds of uterine fibroid prevalence among African Caribbean women in the Tobago Health Study:

Supplemental Materials

Alexis Kiyanda[1], Hoimonty Mazumder[1], Iva Miljkovic[2], Ryan Cvejkus[2], Janet Catov[2], Victor Wheeler [2,3], Patrick J. Parsons[4,5], Elizabeth J. Mullin[4], Austin A. Roberts[4], Aaron Barchowsky[1], Natalie Price[1,2]*, Alison P. Sanders [1,2]*.

[1]Department of Environmental and Occupational Health, University of Pittsburgh School of Public Health, Pittsburgh, PA

[2]Department of Epidemiology, University of Pittsburgh School of Public Health, Pittsburgh, PA

[3]Tobago Health Studies Office, Scarborough, Trinidad & Tobago

[4]Division of Environmental Health Sciences, Wadsworth Center, New York State Department of Health, Albany, NY

[5]Department of Environmental Health Sciences, University at Albany, College of Integrated Health Sciences, Albany, NY

*Authors contributed equally

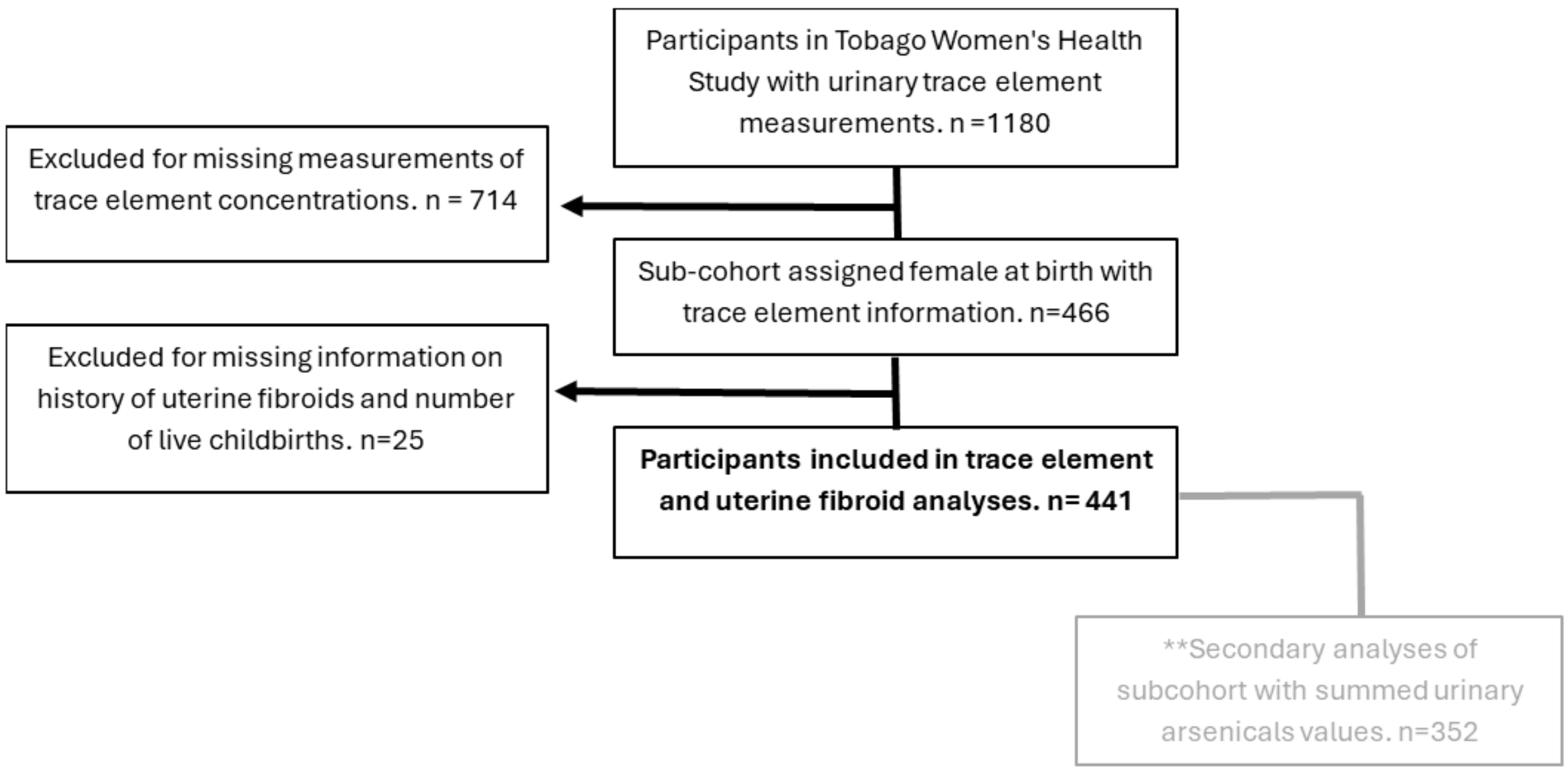

Supplemental Figure 1. Flow chart of THS participant selection in analyses of metals and uterine fibroids.

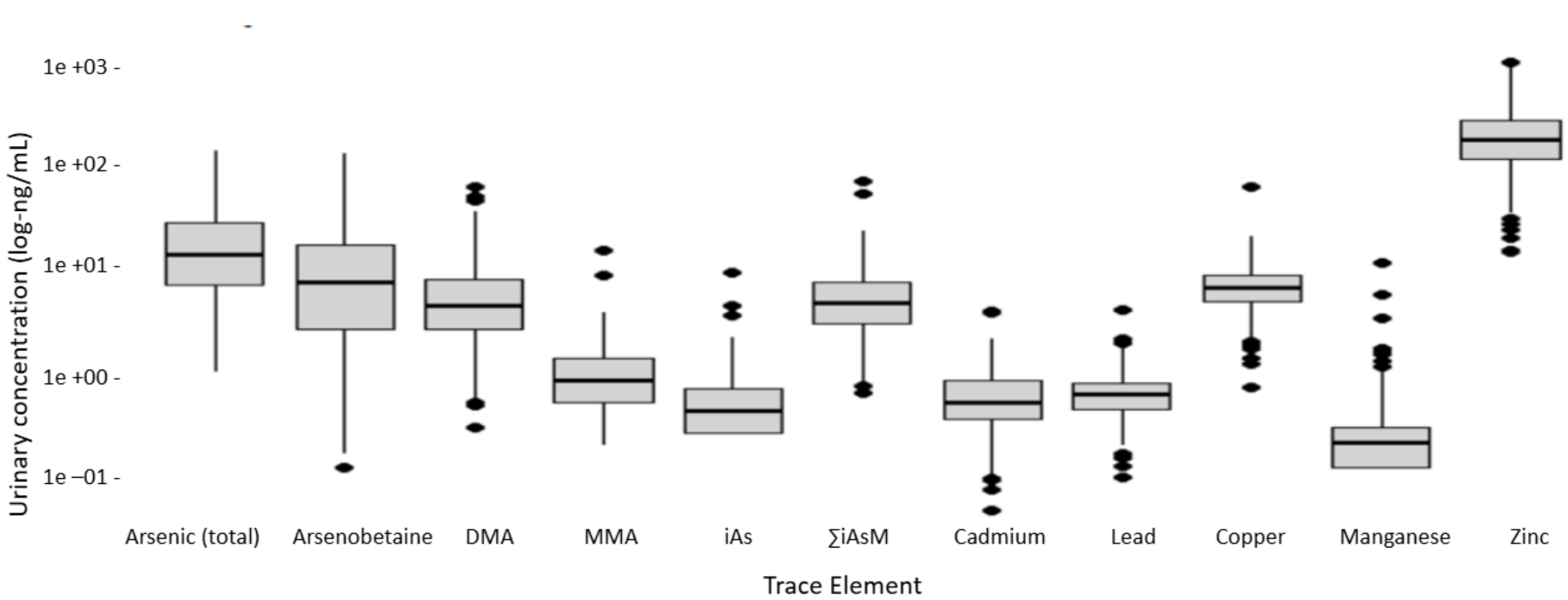

Supplemental Figure 2. Boxplots of urinary metals show relative distributions of each metal.
There are higher concentrations of arsenic and zinc within the cohort of 441 participants.

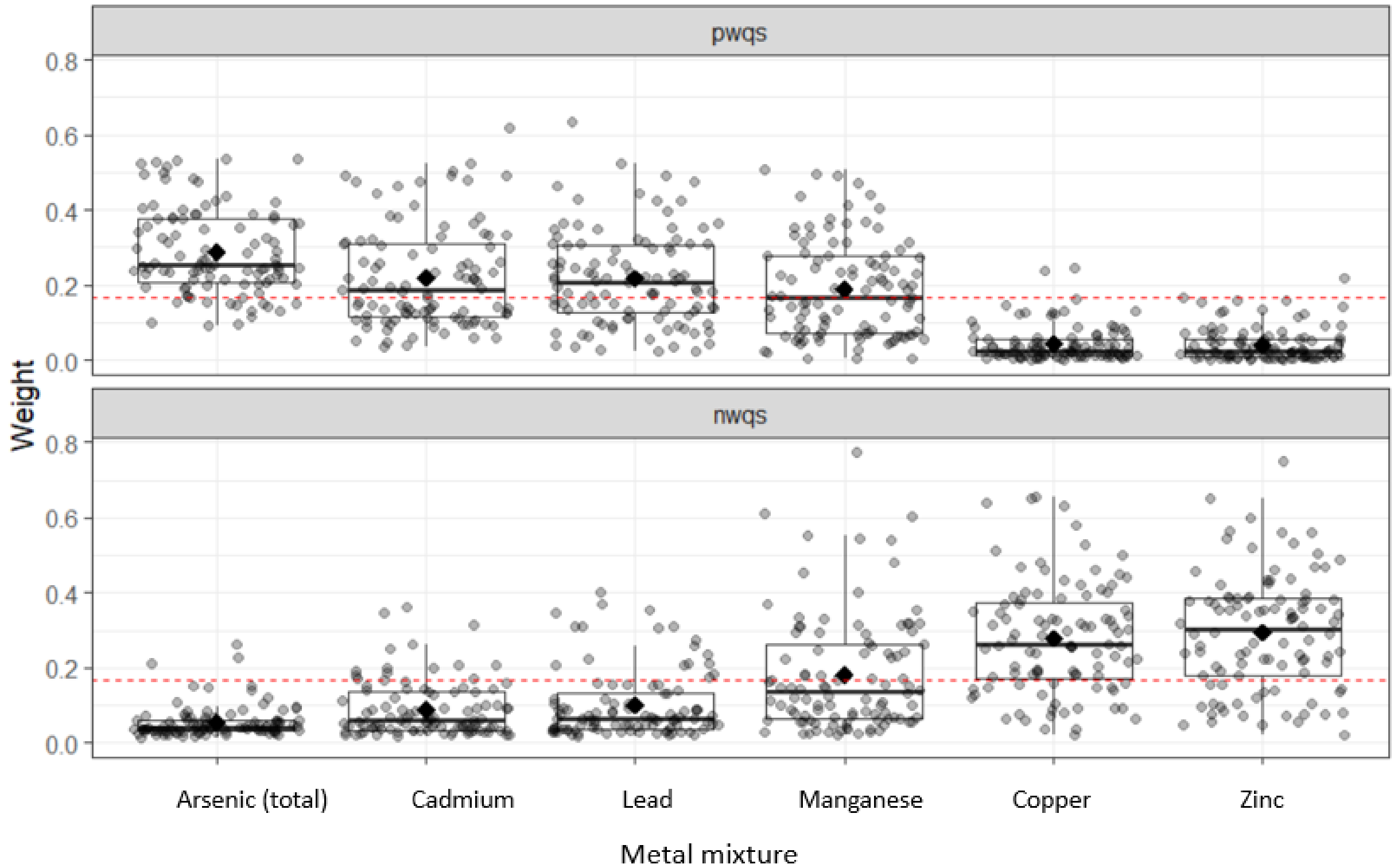

Supplemental Figure 3. gWQS weights by metals in positive (top) and negative (bottom) direction metal mixture models.

The red dotted line corresponds to a weight of 0.16 (1/ the 6 metals). Metals include arsenic, cadmium, lead, manganese, copper and zinc. Arsenic and cadmium had the highest weights in positive direction models, while zinc and copper had the highest weights in the negative direction models.

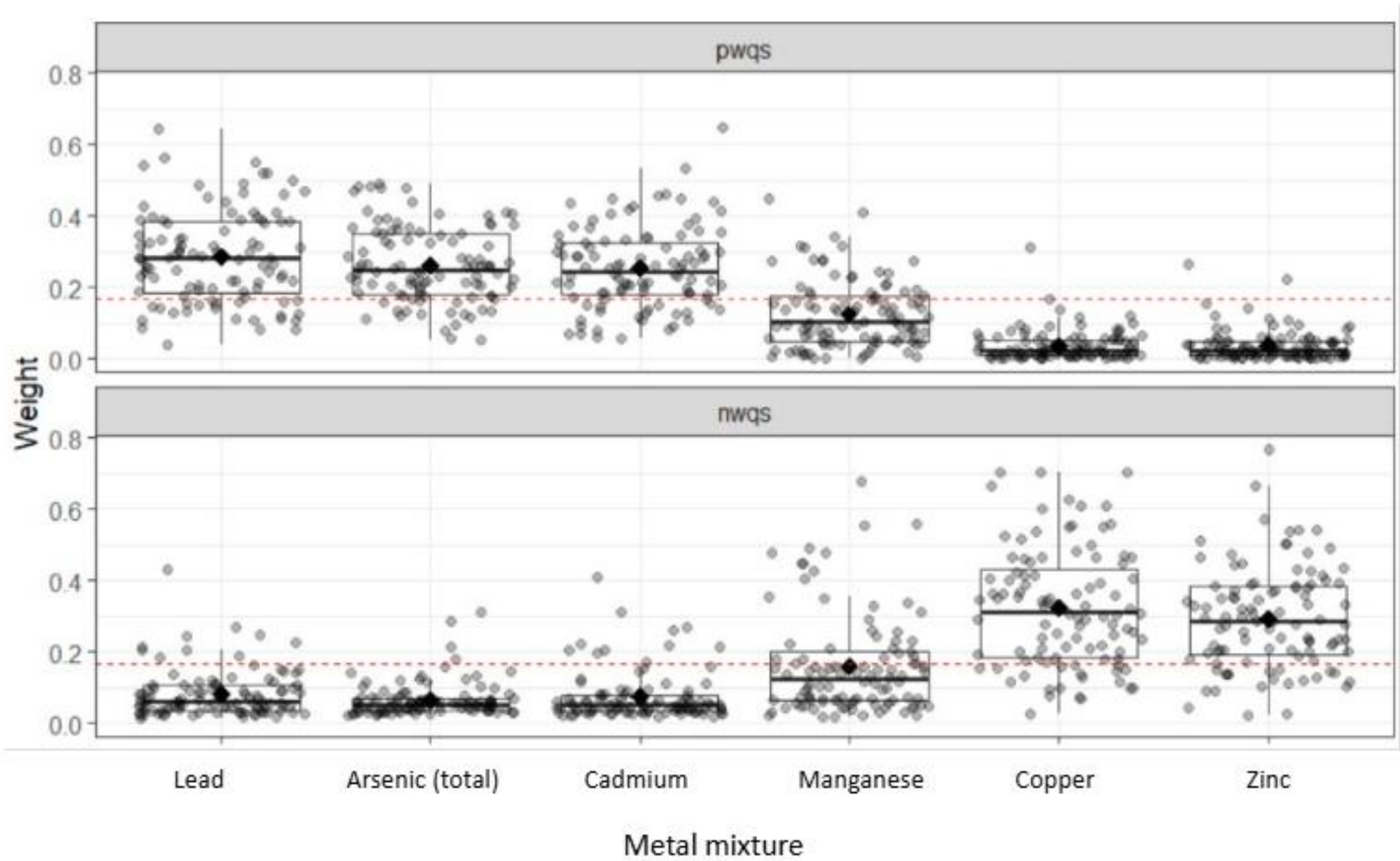

Supplemental Figure 4. gWQS weights by metals in positive (top) and negative (bottom) direction creatinine-standardized metal mixture models.

The red dotted line corresponds to a weight of 0.16 (1/ the 6 metals). Metals include arsenic, cadmium, lead, manganese, copper and zinc. Lead had the highest weights in positive direction models, while zinc and copper had the highest weights in the negative direction models.

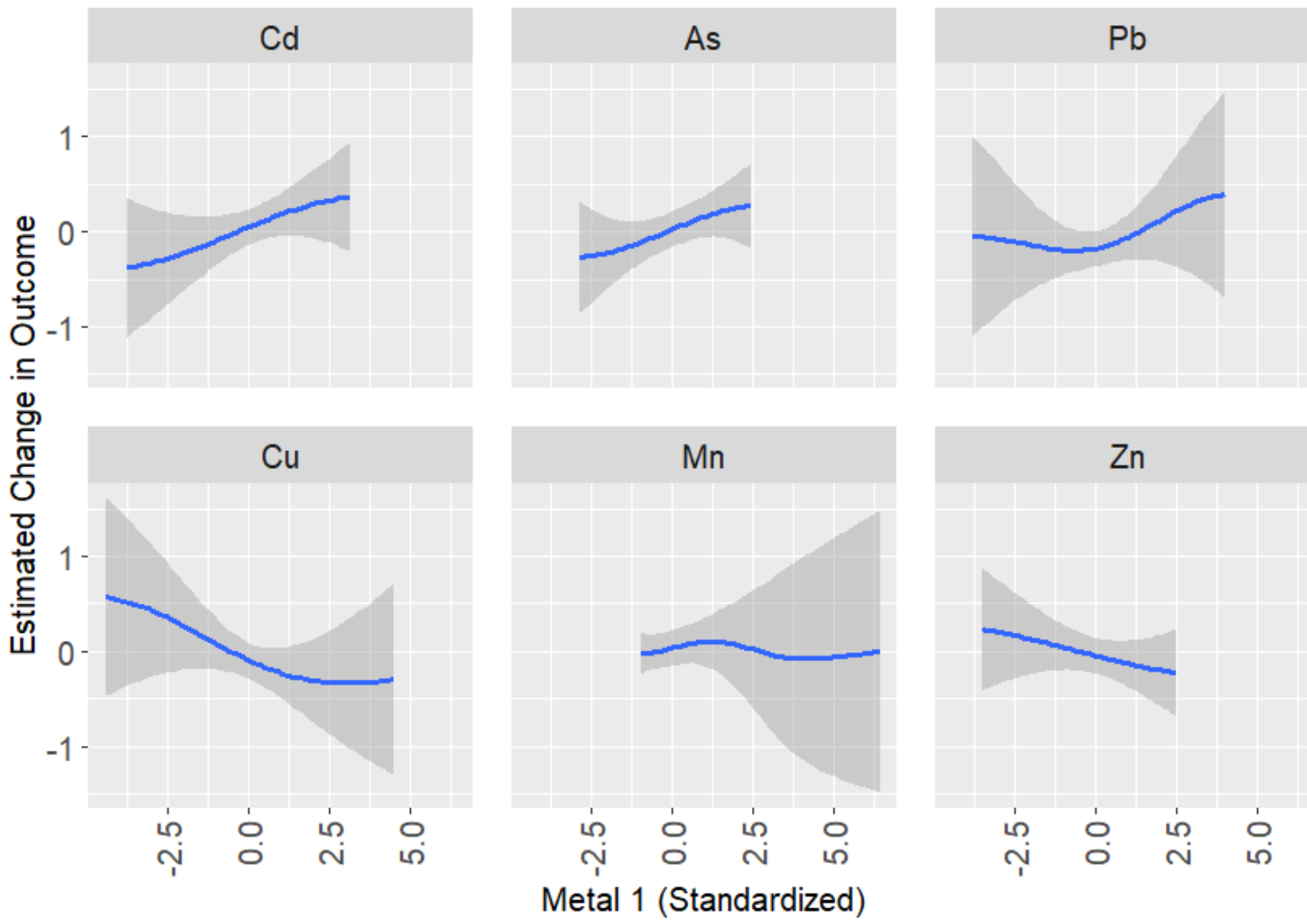

Supplemental Figure 5. BKMR Univariate Exposure-response Functions

Each curve represents the estimated change in odds of uterine fibroids across the exposure range of one metal, holding all other metals at their median values. Shaded areas indicate 95% credible intervals.

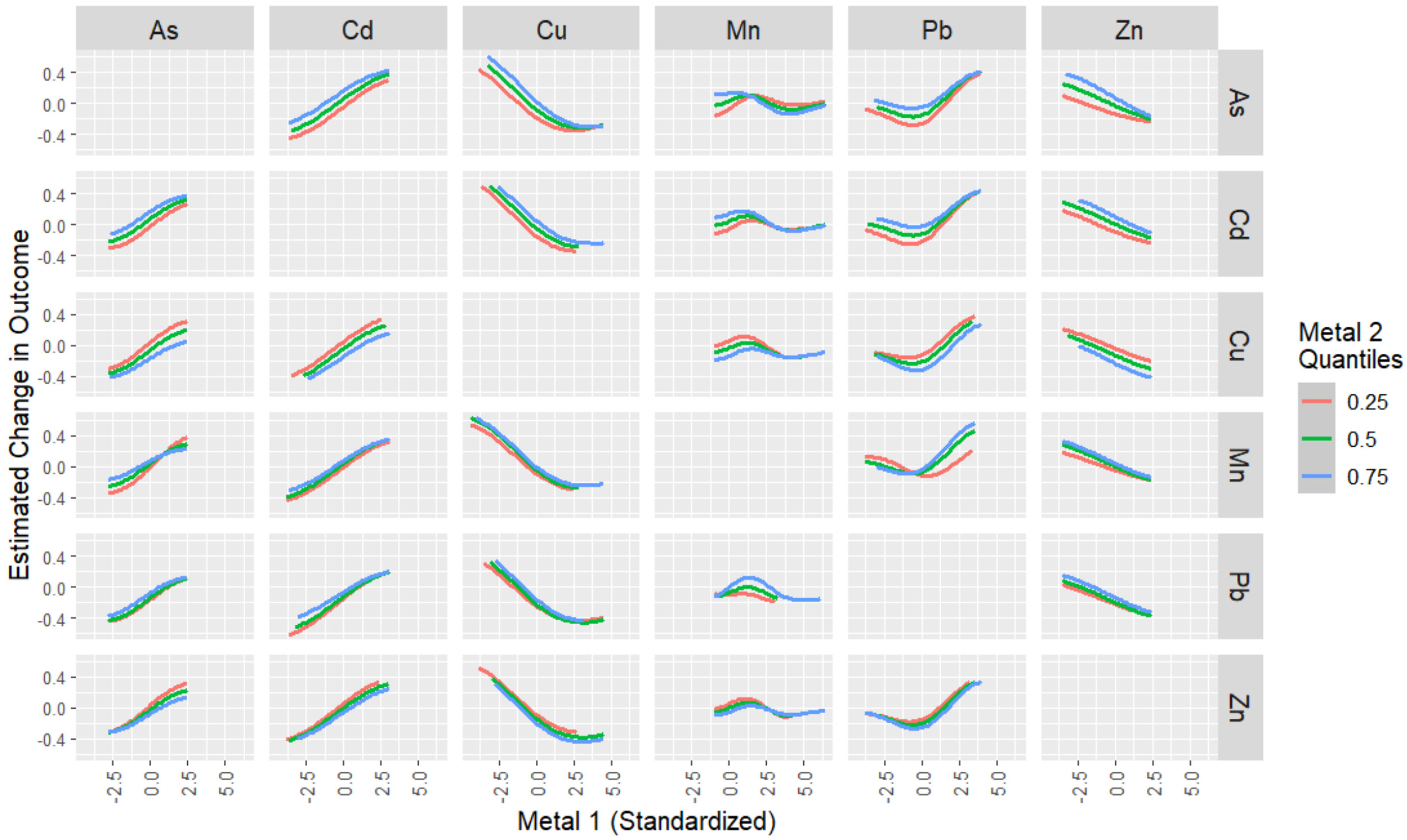

Supplemental Figure 6. BKMR Bivariate Exposure-response Functions

Each curve represents the exposure range of metal 1 on the x-axis and estimated change in the outcome on the y-axis, with metal 2 at the 25th, 50th, and 75th percentile, and all other metals held at their median value.

Supplemental Table 1. Descriptive statistics of urinary metal levels (ug/L) in the Tobago Women's Health Study (n=441).

Metal		Geometric mean (SE)	Median	10th P-tile	25th P-tile	75th P-tile	90th P-tile	Fibroid participants, geometric mean (SE), n = 213	Non-fibroid participants, geometric mean (SE), n = 230
**Non-essential metals**	**Arsenic (total)**	34.7 (2.98)	33.8	9.39	15.3	76.2	146.0	38.5 (2.87)	31.5 (3.06)
	**Arsenobetaine (n=440)[a]**	13.0 (5.60)	16.5	1.40	4.89	43.4	103.1	15.9 (4.99)	10.8 (6.11)
	**DMA (n = 440) [a]**	8.67 (2.82)	8.86	2.60	4.85	17.3	30.4	9.50 (2.76)	7.95 (2.87)
	**MMA (n = 352) [b]**	1.19 (2.42)	1.27	0.24	0.72	2.27	3.41	1.27 (2.27)	1.12 (2.54)
	**iAs (n = 439) [c]**	0.625 (1.96)	0.580	0.325	0.325	1.015	1.54	0.624 (1.91)	0.626 (2.02)
	**∑iAsM [b] (n=352)**	9.02 (2.39)	9.55	2.92	5.74	17.0	27.5	9.56 (2.28)	8.56 (2.49)
	**Cadmium**	0.734 (2.11)	0.727	0.280	0.463	1.28	1.91	0.762 (2.01)	0.708 (2.21)
	**Lead**	0.857 (1.74)	0.886	0.433	0.600	1.20	1.63	0.884 (1.73)	0.833 (1.75)
**Essential metals**	**Copper**	13.8 (1.80)	14.2	6.51	9.93	19.7	28.6	13.3 (1.77)	14.2 (1.82)
	**Manganese**	0.268 (2.05)	0.258	0.134	0.134	0.373	0.603	0.271 (1.97)	0.266 (2.12)
	**Zinc**	627 (2.27)	639	221	392	1060	1850	596 (2.22)	658 (2.31)
**Creatinine concentration in urine (mg/dL)**		149 (1.75)	159	72.4	107	223	278	147 (1.73)	152 (1.76)
**Arsenic methylation efficiency ratio**	**MMA / iAs ***	1.93 (1.76)	2.09	0.74	1.41	2.80	3.65	2.10 (1.71)	1.78 (1.78)
	**DMA / MMA**	5.82 (1.82)	5.64	3.05	4.22	7.82	10.5	5.84 (1.78)	5.80 (1.85)
	**MMA / DMA**	0.171 (1.82)	0.177	0.095	0.128	0.237	0.328	0.171 (1.78)	0.172 (1.85)

[a] Arsenobetaine and DMA were analyzed in a subset of 440 participants with available data. [b] Summed arsenicals and arsenic metabolites of MMA were analyzed in a subset of 352 participants with available data. [c] Inorganic arsenic was analyzed in a subset of 439 participants with available data. Abbreviations: standard error (SE), percentile (P-tile).* $p < 0.05$

Supplemental Table 2. Descriptive statistics of creatinine-standardized urinary metal levels (ug/g) in the Tobago Women's Health Study (n=441).

Metal		Geometric mean (SE)	Median	10th P-tile	25th P-tile	75th P-tile	90th P-tile	Fibroid participants, geometric mean (SE), n = 213	Non-fibroid participants, geometric mean (SE), n = 230
**Non- essential metals**	**Arsenic (total)**	41.2 (55.7)	20.5	6.43	11.3	49.5	95.5	45.2 (57.7)	37.3 (53.6)
	**Arsenobetaine (n=440)[a]**	25.6 (43.0)	10.0	0.913	3.50	27.5	63.9	29.0 (46.5)	22.5 (39.4)
	**DMA (n = 440) [a]**	9.19 (12.8)	5.61	2.00	3.33	10.1	18.4	10.3 (14.2)	8.19 (11.3)
	**MMA (n = 352) [b]**	1.17 (2.29)	0.805	0.315	0.515	1.28	1.82	1.18 (1.50)	1.15 (2.82)
	**iAs (n = 439) [c]**	0.582 (1.12)	0.401	0.187	0.272	0.625	0.967	0.552 (0.653)	0.609 (1.43)
	**∑iAsM [b] (n=352)**	8.70 (14.5)	6.25	2.48	3.86	9.03	13.9	9.15 (14.9)	8.29 (14.3)
	**Cadmium**	0.589 (0.408)	0.498	0.235	0.331	0.709	0.985	0.586 (0.408)	0.586 (0.408)
	**Lead**	0.690 (0.563)	0.553	0.287	0.401	0.802	1.11	0.738 (0.658)	0.645 (0.454)
**Essential metals**	**Copper**	10.4 (7.25)	8.77	6.07	0.073	0.113	0.143	10.2 (6.92)	10.5 (7.81)
	**Manganese**	0.293 (0.601)	0.159	0.071	0.105	0.277	0.502	0.285 (0.474)	0.298 (0.700)
	**Zinc**	538 (503)	414	193	284	617	966	520 (458)	556 (542)
**Arsenic methylation efficiency ratio**	**MMA / iAs ***	1.93 (1.76)	2.09	0.74	1.41	2.80	3.65	2.10 (1.71)	1.78 (1.78)
	**DMA / MMA**	5.82 (1.82)	5.64	3.05	4.22	7.82	10.5	5.84 (1.78)	5.80 (1.85)
	**MMA / DMA**	0.171 (1.82)	0.177	0.095	0.128	0.237	0.328	0.171 (1.78)	0.172 (1.85)

[a] Arsenobetaine and DMA were analyzed in a subset of 440 participants with available data. [b] Summed arsenicals and arsenic metabolites of MMA were analyzed in a subset of 352 participants with available data. [c] Inorganic arsenic was analyzed in a subset of 439 participants with available data. Abbreviations: standard error (SE), percentile (P-tile).

Supplemental Table 3. Adjusted odds ratios of uterine fibroid prevalence in adjusted models and models with individual creatinine-standardized urinary metals.

Metal		Adjusted Model OR (95% CI), n = 441	Creatinine-standardized Model OR (95% CI), n = 441
**Non-essential metals**	**Arsenic (total)**	1.61 (1.04, 2.53)*	1.63 (1.05, 2.55)*
	**Arsenobetaine[a]**	1.38 (1.05, 1.82)*	1.38 (1.06, 1.83)*
	**DMA[a]**	1.84 (1.11, 3.09)*	1.85 (1.12, 3.11)*

	**MMA[b]**	1.99 (0.99, 4.02)	2.00 (1.02, 4.02)*
	**iAs[c]**	1.11 (0.54, 2.28)	1.20 (0.62, 2.31)
	**∑iAsM[b]**	1.89 (0.93, 3.90)	1.90 (0.95, 3.91)
	**Cadmium**	2.03 (0.90, 4.63)	2.06 (0.92, 4.69)
	**Lead**	2.42 (0.95, 6.31)	2.20 (0.98, 5.03)
**Essential metals**	**Copper**	0.55 (0.18, 1.67)	0.65 (0.22, 1.86)
	**Manganese**	1.11 (0.59, 2.09)	1.18 (0.69, 2.03)
	**Zinc**	0.67 (0.34, 1.31)	0.69 (0.35, 1.35)

[a] Arsenobetaine and DMA were analyzed in a subset of 440 participants with available data. [b] Summed arsenicals and arsenic metabolites of MMA were analyzed in a subset of 352 participants with available data. [c] Inorganic arsenic was analyzed in a subset of 439 participants with available data. * Metals and metabolites associated with uterine fibroids with p<0.05.

Supplemental Table 4. Odds ratios of non-essential and essential metals and analyte weights in bidirectional WQS model

		**WQS Index**			
**Analyte**		**Positive WQS Index**		**Negative WQS Index**	
		**OR and CI:** 1.33 [0.92, 1.93]		**OR and CI:** 0.95 [0.62, 1.44]	
		**metal weights**	**CI [2.5%, 97.5%]**	**metal weights**	**CI [2.5%, 97.5%]**
**Non-essential metals**	**Arsenic**	0.29	[0.11, 0.53]	0.06	[0.02, 0.18]
	**Cadmium**	0.22	[0.05, 0.50]	0.09	[0.02, 0.29]
	**Lead**	0.22	[0.03, 0.44]	0.10	[0.02, 0.35]
**Essential metals**	**Copper**	0.04	[0.00, 0.16]	0.28	[0.05, 0.64]
	**Manganese**	0.19	[0.01, 0.48]	0.18	[0.02, 0.58]
	**Zinc**	0.04	[0.00, 0.16]	0.29	[0.05, 0.58]

Weights of individual metals within gWQS and nWQS models. CI = confidence intervals. Arsenic contributed the highest weight in the positive model of increased odds of uterine fibroid prevalence.

Supplemental Table 5. Odds ratios of creatinine-standardized urinary non-essential and essential metals and analyte weights in bidirectional WQS model

	**WQS Index**	
**Analyte**	**Positive WQS Index**	**Negative WQS Index**

		**OR and CI:** 1.43 [0.90, 2.27]		**OR and CI:** 0.95 [0.84, 1.28]	
		**metal weights**	**CI [2.5%, 97.5%]**	**metal weights**	**CI [2.5%, 97.5%]**
**Non-essential metals**	**Arsenic**	0.26	[0.09, 0.48]	0.07	[0.02, 0.20]
	**Cadmium**	0.25	[0.07, 0.46]	0.07	[0.02, 0.26]
	**Lead**	0.29	[0.08, 0.55]	0.08	[0.02, 0.24]
**Essential metals**	**Copper**	0.04	[0.00, 0.13]	0.32	[0.07, 0.69]
	**Manganese**	0.12	[0.00, 0.33]	0.16	[0.02, 0.52]
	**Zinc**	0.04	[0.00, 0.15]	0.29	[0.06, 0.56]

Weights of individual metals within gWQS and nWQS models. CI = confidence intervals. Lead contributed the highest weight in the positive model of increased odds of uterine fibroid prevalence.
